# Chemical Constituents of the Essential Oil from Ecuadorian Endemic Species *Croton ferrugineus* and Its Antimicrobial, Antioxidant and α-Glucosidase Inhibitory Activity

**DOI:** 10.3390/molecules26154608

**Published:** 2021-07-29

**Authors:** Eduardo Valarezo, Génesis Gaona-Granda, Vladimir Morocho, Luis Cartuche, James Calva, Miguel Angel Meneses

**Affiliations:** Departamento de Química, Universidad Técnica Particular de Loja, Loja 110150, Ecuador; gensg_64@hotmail.com (G.G.-G.); svmorocho@utpl.edu.ec (V.M.); lecartuche@utpl.edu.ec (L.C.); jwcalva@utpl.edu.ec (J.C.); mameneses@utpl.edu.ec (M.A.M.)

**Keywords:** *Croton ferrugineus*, essential oil, chemical composition, antiglucosidase activity, antimicrobial activity, antioxidant activity

## Abstract

*Croton ferrugineus* Kunth is an endemic species of Ecuador used in traditional medicine both for wound healing and as an antiseptic. In this study, fresh *Croton ferrugineus* leaves were collected and subjected to hydrodistillation for extraction of the essential oil. The chemical composition of the essential oil was determined by gas chromatography equipped with a flame ionization detector and gas chromatography coupled to a mass spectrometer using a non-polar and a polar chromatographic column. The antibacterial activity was assayed against three Gram-positive bacteria, one Gram-negative bacterium and one dermatophyte fungus. The radical scavenging properties of the essential oil was evaluated by means of DPPH and ABTS assays. The chemical analysis allowed us to identify thirty-five compounds representing more than 99.95% of the total composition. Aliphatic sesquiterpene hydrocarbon trans-caryophyllene was the main constituent with 20.47 ± 1.25%. Other main compounds were myrcene (11.47 ± 1.56%), β-phellandrene (10.55 ± 0.02%), germacrene D (7.60 ± 0.60%), and α-humulene (5.49 ± 0.38%). The essential oil from *Croton ferrugineus* presented moderate activity against *Candida albicans* (ATCC 10231) with an MIC of 1000 μg/mL, a scavenging capacity SC_50_ of 901 ± 20 µg/mL with the ABTS method, and very strong antiglucosidase activity with an IC_50_ of 146 ± 20 µg/mL.

## 1. Introduction

The Euphorbiaceae family comprises approximately 6547 species belonging to 228 plant genera [1]. The Euphorbiaceae species are herbs, shrubs, and trees of flowering plants (order Malpighiales). Many members are important food sources. Others are useful for their waxes and oils and as a source of medicinal drugs, dangerous for their poisonous fruits, leaves, or sap; or attractive for their colorful bracts (leaflike structures located just below flower clusters) or unusual shapes [2]. It is a cosmopolitan family distributed throughout the world, with the exception of the polar areas, being better represented in tropical and subtropical areas [3]. This family occurs mainly in the tropics, with most of the species distributed throughout the Indo-Malay region and tropical America. There is a wide variety in tropical Africa, although not as abundant or varied as in the other two regions. However, genus Euphorbia also has many species in non-tropical areas, such as the Mediterranean Basin, the Middle East, South Africa, and southeastern United States [4].

Genus *Croton*, with approximately 1205 species [1], is the most diverse of the Euphorbiaceae family worldwide and, though native to South America, it presents global distribution [5] and is found in a wide variety of habitats in tropical and subtropical regions of both hemispheres [6]. *Croton* species are known as traditional medicinal plants in Africa, Asia, and South America [7]. Several of these plants are used in folk medicine as cicatrizing, anti-inflammatory, anticancer, against gastric problems, to treat hemorrhoids, and as agents to control uterine hemorrhages [8]. In *Croton* spp., the presence of secondary metabolites has been reported, as it is latex rich mainly in alkaloids, flavonoids, terpenes, terpenoids and ricin-type toxins [9]; due to the presence of these bioactive compounds, the *Croton* species are commonly used to treat cancer, hypertension, inflammation, rheumatism, bleeding gums, malaria, asthma, syphilitic ulcers, diabetes, pain, and ulcers [7]. The traditional uses of *Croton* spp. have frequently been confirmed by pharmacological assays. *Croton* extracts and isolated compounds reported antioxidant, antihypertensive, antimalarial, antimicrobial, antispasmodic, antiulcer, antiviral, myorelaxant, cytotoxic, anti-inflammatory, neuroprotective, antitumor, anticancer, insecticidal, and allelopathic activity [8,10]. Several species of genus *Croton* are aromatic, indicating the presence of volatile oil constituents. The essential oil from *Croton* spp. has been reported with anti-inflammatory, antinociceptive, gastroprotective, antileishmanial, antimicrobial, anti-gastric ulcer and antiparasitic activity, as well as cardiovascular, intestinal myorelaxant, and antispasmodic effects [8].

In Ecuador, 244 species have been recorded for the Euphorbiaceae family, of which 46 are endemic. In addition, 46% of the endemic taxa of this group grow in foothill forests and inter-Andean vegetation, 11 live in coastal forests; nine species are restricted to the Galapagos, and another three species grow in the Galapagos and on the mainland. The genus with the highest number of endemics in the country is *Croton*, with 13 species [1].

*Croton ferrugineus* Kunth is an endemic shrub of Ecuador; in addition, it can be found in the West and North of South America, especially in Colombia, Peru, and Brazil [11]. This species is distributed from humid forests to xerophytic zones between 500–2000 m a.s.l. [12]. In Ecuador, it can be found in the Andean province of Loja [13]. The plant is popularly known as “Mosquera”. The *Croton ferrugineus* species is used in traditional medicine for wound healing; in Colombia, it is employed to treat thornback ray (*Raja clavata*) bites and as an antiseptic [14].

The facts that the secondary metabolites extracted from *Croton* spp. present potential application in food, cosmetics, pharmaceuticals, and agrochemical products [10]. The essential oils isolated from these species reporting different biological activities, and the study of the *C. ferrugineus* essential oil having not been previously reported in the literature have stimulated our interest in investigating the essential oil extracted from this species. For that reason, the objective of this research is to determine the chemical composition and antimicrobial, antioxidant, and α-Glucosidase inhibitory activity of the *C. ferrugineus* essential oil. To our knowledge, this is the first report of the study of the *C. ferrugineus* essential oil.

## 2. Results

Approximately 17,000 g of aerated leaves, 42 ± 2% moisture, of *C. ferrugineus* were subjected to hydrodistillation; in total (nine samples: three distillations × three collections), 10 mL of essential oil were obtained, which represents a yield of 0.06 ± 0.02% (*v*/*w*) or 0.6 mL/kg, which is considered as low yield [15]. The essential oil was a viscous liquid of subjective light-yellow color (RGB values of R:255, G:255, B:237, and CMYK values of C:0, M:0, Y:0.07, K:0). The density of the essential oil was *d*^20^ = 0.9233 ± 0.0098 g/cm^3^, its refractive index was *n*^20^ = 1.4850 ± 0.0011, and the specific rotation was [α]_D_^20^ = −16.6 ± 0.1° (levo-rotatory).

The identification of volatile compounds present in the *C. ferrugineus* EO was carried out by GC/MS and GC/FID. The qualitative and quantitative data of the chemical composition obtained using nonpolar column DB―5 ms are shown in Table 1. Thirty-five compounds were identified, which represent 99.95% of the total composition. The oil compounds were mainly grouped into aliphatic sesquiterpene hydrocarbons (ALS) with a representation of 43.50%. In addition, the compounds grouped as aliphatic monoterpene hydrocarbons (ALM) represented 34.98% and compounds belonging to the aromatic sesquiterpene hydrocarbons (ARS) group were not identified. The ALS trans-caryophyllene (compound 20, CF: C_15_H_24_, MM: 204.19 Da) was the main constituent with 20.47 ± 1.25%. Other main compounds (>5%) were myrcene (511.47 ± 1.56%), germacrene D (257.60 ± 0.60%), and α-humulene (235.49 ± 0.38%). Compounds 9 and 10 (limonene and β-phellandrene) eluted together (co-eluted), both representing 14.88 ± 1.27%.

An HP-INNOWax polar column was used to separate, identify, and determine the percentage of coeluted compounds (limonene and β-phellandrene). The RIs in the polar column were 1194 for limonene and 1204 for β-phellandrene. Limonene presented a percentage of 4.74 ± 0.03% and β-phellandrene a value of 10.55 ± 0.02%.

The essential oils from *C. ferrugineus* leaves were assessed by the microdilution broth method against Gram-negative bacteria, Gram-positive bacteria, and dermatophyte Fungi; the minimum inhibitory concentration (MIC, μg/mL) values are shown in Table 2. At the maximum evaluated concentration of 2500 µg/mL, the EOs reported MIC values of 1000 µg/mL against *Cándida albicans* (ATCC 10231) and of 2000 µg/mL against *Micrococcus luteus* (ATCC 10240).

The essential oil from *C. ferrugineus* was explored for antioxidant activity using DPPH^•^ and ABTS^•+^ scavenging activity. Trolox was used as a positive control, and the results obtained are shown in Table 3. SC_50_ was used as a measure of the inhibition concentration value of 50% of the activity. Employing the ABTS technique, the EO showed SC_50_ = 901 ± 20 μg/mL. Through the DPPH method, essential oils do not provide SC_50_ values at the maximum concentration tested of 1000 μg/mL.

The *C. ferrugineus* essential oil was assessed for its antiglucosidase potential; the results obtained are shown in Table 3. The IC_50_ value obtained for the *C. ferrugineus* EO was 146 ± 20 µg/mL. Positive control acarbose presented an IC_50_ value of 70 ± 10 μg/mL.

## 3. Discussion

The essential oil from C. *ferrugineus* showed a low extraction yield of 0.09 ± 0.02% From the chemical composition, eight components were identified as main compounds: trans caryophyllene (20.47%), myrcene (11.47%), β-phellandrene (10.55%), germacrene D (7.60%), linalool (7.34%), and α-humulene (5.49%); the compounds are grouped predominantly as sesquiterpenes (43.5%) and monoterpenes (34.98%). This is the first report of the chemical composition of the essential oil from *C. ferrugineus*.

However, the composition of other *Croton* genus species can be considered to compare the main components. Lima et al. [8] studied the essential oil from *C. adenocalyx*, and the main components reported were α-pinene (32.63%), bicyclogermacene (13.96%), trans caryophyllene (10.23%), germacrene D (10.14%), β-pinene (10.11%), and β-elemene (8.31%). Similarly, de Araujo et al. [16] reported the chemical composition of the essential oil from *C. ceanothifolius* and the main compounds were bicyclogermacrene (26.3%), germacrene D (14.7%), (E)-caryophyllene (11.7%), 1,10-di-epi-cubebol (7.4%), germacrene A (4.7%), and β-elemene (4.2%).

The main component (trans caryophyllene) has been reported as frequent in *Croton* spp. [8]; in addition, germacrene D and bicyclogermacrene were also identified in different reports of the EO from *Croton* spp. The chemical composition of the *C. ferrugineus* EO indicates predominance of sesquiterpenes and, according to de Araújo et al. [16], *Croton* spp. presents similarities in the main components.

Trans caryophyllene is a sesquiterpene that has demonstrated anti-spasmodic [17] and antileishmanial activity [18]. Myrcene is a terpene studied for cardioprotective, anti-inflammatory, and antioxidant effects [19]. On the other hand, Stević et al. [20] analyzed the biological activity of linalool and determined a very strong antibacterial activity and strong antifungal activity. Germacrene D has proved to have high antibacterial and antifungal activity [21].

Regarding antimicrobial activity, the essential oil from *C*. *ferrugineus* showed no activity against the pathogenic microorganism tested (MIC > 2000 µg/mL). MIC was 1000 µg/mL only against *Candida albicans*. Considering the classification of the EO’s biological activity expressed by van Vuuren and Holl [22], MIC values between 500 and 1000 µg/mL are reported as moderate activity. In this case, the EO from *C. ferrugineus* is poor compared to the activity of the main chemical components in the EO from *C. ferrugineus*, which have been reported with strong antibacterial and antifungal activity; for example, Dahham et al. [23] reported that trans caryophyllene (synonymous β-caryophyllene) was very effective against different bacterial and fungal strains. These differences could be due to the fact that EOs are a complex chemical mixture that produces synergistic or antagonistic responses. Compared to data reported in the literature, de Araújo et al. [16] obtained MIC results greater than 1024 μg/mL, indicating that it is not clinically relevant. On the other hand, de Araújo et al. [24] reported that the EO from *C. heliotropiifolius* Kunth presented inhibitory activity against *Bacillus subtilis* and *Staphylococcus aureus*, and de Araújo et al. [25] reported inhibitory activity against *Staphylococcus aureus* by the EO from *C. argyrophyllus*.

The antioxidant properties represent the first stage of evaluation of health properties and, similarly to other activities, the antioxidant efficacy results from the combination of chemical bioactive compounds. The antioxidant efficacy of the main component, trans caryophyllene, has been reported by Dahham et al. [23] as SC_50_ with a value of 1.25 ± 0.06 μM. The antioxidant activity of the EO from *C. ferrugineus* showed SC_50_ values of 901 ± 20 for ABTS and >1000 for DPPH assays. Morais et al. [26] reported a moderate antioxidant activity of the EO from four C. species, expressed as SC_50_ in μg/mL, *C. argyrophylloides* 12.55 ± 0.43, *C. jacobinensis* 22.11 ± 3.24, *C. nepetifolius* 24.96 ± 6.87, and *C. sincorensis* 80.59 ± 18.98. Rossi et al. [27] reported lower SC_50_ for the EO from *C. lechleri*.

The EO from *C. ferrugineus* presents a strong inhibitory effect of α-glucosidase with IC_50_ 146 ± 20 μg/mL, although the result for the acarbose positive control was 70 ± 10 μg/mL. Data about the α-glucosidase inhibitory activity of the EO are limited. Jelassi et al. [28] analyzed the α-glucosidase inhibitor activity of the EOs from *Acacia mollisima* and *Acacia cyclops*, which present strong inhibition of this enzyme with IC_50_ values of 81–89 μg/mL. Bensouici et al. [29] analyzed the EO from *Rosmarinus tournefortii* and did not report α-glucosidase inhibitor activity at the highest concentration of 500 μg/mL. Morocho et al. [30] reported the α-glucosidase inhibition of the chemical constituents of *C. thurifer* Kunth, although these correspond to non-volatile compounds. Due to the hypoglycemic activity of the EO from *C. ferrugineus*, it can be considered as a novel α-glucosidase inhibitor. This result is attributed to the complex mixture of the EO, and it should be necessary to analyze the bioactivity of the main chemical compounds and to perform further toxicity studies.

## 4. Materials and Methods

### 4.1. Materials

Sodium sulfate anhydrous, dichloromethane (DCM), methanol (MeOH), 6-hydroxy-2,5,7,8-tetramethylchroman-2-carboxylic acid (Trolox), 2,2-diphenyl-1-picrylhydryl (DPPH), 2,2′-azinobis-3-ethylbenzothiazoline-6-sulfonic acid (ABTS), phosphate buffered saline (PBS, SIGMA-P4417), and p-Nitrophenyl-α-d-glucopyranoside (pNPG) were purchased from Sigma-Aldrich (San Luis, MO, USA). Dimethylsulfoxide (DMSO) was obtained from Fisher (Hampton, NH, USA). The microbiological media such as Sabourad and Mueller Himton Broth were purchased from DIPCO (Quito, Ecuador). The aliphatic hydrocarbons standard was purchased from CHEM SERVICE (West Chester, PA, USA). Helium was purchased from INDURA (Quito, Ecuador). All chemicals were of analytical grade and used without further purifications.

### 4.2. Plant Material

The leaves of *C. ferrugineus* were collected in mid-June in the surroundings of the locality of Catamayo, province of Loja, Ecuador, at 1400 m a.s.l. and at a latitude of 3°58′57″ S and a longitude of 79°19′47″ W. Collection of the species was performed with authorization No. 001-IC-FLO-DBAP-VS-DRLZCH-MA of the Ecuadorian Environmental Ministry (*Ministerio del Ambiente de Ecuador*, MAE). The plant material was identified by botanist Nixon Cumbicus, and voucher specimens were deposited at the HUTPL Herbarium of *Universidad Técnica Particular de Loja*. Storage and transfer of the plant material were carried out in airtight plastic containers until its use. Collection was at room temperature (22–24 °C) and the transfer temperature was 20–22 °C; pressure was approximately 90 KPa (room pressure).

### 4.3. Postharvest Treatments

The postharvest treatments were implemented immediately after the plant material arrived at the laboratory, between 1 and 2 h after being collected, and consist of the separation of foreign material and degraded leaves.

### 4.4. Moisture Determination

The moisture of the plant material was determined using test method AOAC 930.04–1930, Loss on drying (Moisture) in plants. For moisture determination, an analytical scale (Mettler AC 100, Columbus, OH, USA) was used.

### 4.5. Essential Oil Extraction

For the extraction of the essential oil, the plant material was hydrodistilled for 4 h in a Clevenger-type device, for which a sample mixed with water, in a 1:5 relation, was boiled to evaporate volatile components; then, two layers (aqueous and oil-rich) were obtained and the oil was separated via a separating funnel. Subsequently, the moisture in the essential oil collected was removed by adding anhydrous sodium sulphate and, finally, it was stored in amber sealed vials at 4 °C to protect it from the light until being used in the subsequent analysis composition [31].

### 4.6. Determination of the Physical Properties of the Essential Oil

Density was determined using the AFNOR NF T 75-111 standard (equivalent to the ISO 279:1998 standard) and, for the refractive index, the AFNOR NF T 75–112 standard [32] (similar to ISO 280:1998) was used. Density was measured using an analytical scale (Mettler AC 100 model, ±0.0001) and a pycnometer (1 mL) and, for the refraction index, a refractometer (model ABBE) was used. The measurements were taken at 20 °C. Optical rotation was determined using an automatic polarimeter model Mrc-P810 according to the ISO 592:1998 standard method.

### 4.7. Identification of the Chemical Constituents of the Essential Oil

#### 4.7.1. Quantitative Analysis

For the quantitative analysis, an Agilent gas chromatograph (GC) (model 6890N series, Agilent Technologies, Santa Clara, CA, USA) equipped with a flame ionization detector (FID) was used. The GC-FID analyses were performed using a nonpolar Agilent J&W DB-5ms Ultra Inert GC column (30 m, 0.25 mm, 0.25 µm) (5%-phenyl-methylpolyxilosane), a polar Agilent J&W HP-INNOWax GC column (30 m, 0.25 mm, 0.25 µm) (polyethylene glycol), and an automatic injector (Agilent 7683 automatic liquid sampler, Agilent Technologies, Santa Clara, CA, USA) in split mode. The samples, 1 µL of solution (1/100, *v*/*v*, EO/DCM), were injected with a split ratio of 1:50. Helium was used as a carrier gas at 1 mL/min in constant flow mode and an average speed of 25 cm/s. The initial oven temperature was maintained at 50 °C for 3 min. and then heated to 230 °C with a ramp of 3 °C/min, and the temperature was maintained for 3 min until the end. The injector and detector temperatures were 250 °C. Quantification was done by the external standard method using calibration curves generated by running a GC analysis of representative compounds.

#### 4.7.2. Qualitative Analysis

For the qualitative analysis, an Agilent gas chromatograph (model 6890N series, Agilent Technologies, Santa Clara, CA, USA) was used, coupled to a mass spectrometer (quadrupole) detector (MS) (model Agilent 5973 inert series, Agilent Technologies, Santa Clara, CA, USA). The GC-MS analyses were performed using the same columns and injector as GC-FID. The samples were injected with a split ratio of 1:50. Helium was used as a carrier gas at 0.9 mL/min in constant flow mode and an average speed of 34 cm/s. The operating conditions for the MS were as follows: electron multiplier 1670 eV, 70 eV, mass range 45–350 *m*/*z*, and scan rate 2 scan/s. The MS was provided with an MSD-Chemstation D.01.00 SP1 computerized system. Identification of the oil components was based on a comparison of mass spectrum data with the Wiley 7n libraries from the internal chromatograph database, and mass spectrum data and relative retention indices (RIs) with those of the published literature [33,34]. The RIs of the compounds were determined based on the homologous of the standard aliphatic hydrocarbons, which were injected after the oils in the same conditions. The RIs were obtained through the arithmetic index described by van Den Dool and Dec. Kratz [23] using Equation (1):

(1) $$\mathrm{RI}=100n+\frac{\left(\mathrm{RTx}-\mathrm{RTn}\right)}{\left(\mathrm{RTN}-\mathrm{RTn}\right)}$$

where n is the carbon number of the hydrocarbon that elutes before the compound of interest, RTx is the retention time (RT) of the compound of interest, RTn is the RT of the hydrocarbon that elutes before the compound of interest, and RTN is the RT of the hydrocarbon that elutes after the compound of interest.

### 4.8. Evaluation of the Antimicrobial Activity

#### 4.8.1. Antibacterial Activity

The antibacterial activity of the essential oil was determined against three Gram-negative bacteria and one Gram-positive bacterium (Table 2) according to the procedure described by Valarezo et al. [35]. The bacterial strains were incubated in Müeller-Hinton (MH) broth, and DMSO was used to dissolve the essential oil. Tetracycline and gentamicin were used as a positive control. DMSO was used as a negative control, and the results are reported as minimum inhibitory concentration (MIC) (the lowest concentration of sample capable of inhibiting all visible signs of growth of the microorganism).

#### 4.8.2. Antifungal Activity

The antifungal activity of the essential oils was determined against a dermatophyte fungus organism (Table 2) by the microdilution method according to the procedure previously described by Valarezo et al. [35]. Ketoconazole was used as a positive control, and DMSO was used as a negative control. The results are reported as minimum inhibitory concentration (MIC).

### 4.9. Antioxidant Capacity

#### 4.9.1. DPPH Radical Scavenging Activity

The 2,2-diphenyl-1-picrylhydryl (DPPH^•^) free radical scavenging activity of the essential oils was measured based on the technique by Brand Williams et al. [36] with some modifications as detailed by Thaipong et al. [37], as previously described by Valarezo et al. [38]. The concentrations of the essential oil from *C. ferrugineus* used were 25, 150, 300, 450, 600, 800, and 1000 ppm [35]. The samples were evaluated at a wavelength of 515 nm in a UV spectrophotometer (Genesys 10S UV.Vis Spectrophotometer, Thermo Scientific, Waltham, MA, USA). Trolox were used as a positive control and methanol as a blank control. The scavenging capacity (SC) was expressed as a percentage and was calculated according to Equation (2):

(2) $$\mathrm{SC}\left(\%\right)=\frac{\left(\mathrm{As}-\mathrm{Ai}\right)}{\mathrm{As}}\times 100$$

where Ai is the absorbance of DPPH^•^ mixed with the EO and As is the sample blank absorbance of DPPH^•^ in which the EO has been substituted with methanol. SC(%) is the EO concentration that provided a 50% DPPH^•^ scavenging effect.

#### 4.9.2. ABTS Radical Cation Scavenging Activity

The ABTS assay was performed according to the procedure report by Arnao et al. [39] with some modifications by Thaipong et al. [37], as previously described by Valarezo et al. [38], using 2,2′-azinobis-3-ethylbenzothiazoline-6-sulfonic acid radical cation (ABTS^•+^). The concentrations of the essential oil from *C. ferrugineus* used were 25, 150, 300, 450, 600, 800, and 1000 ppm. The samples were evaluated at a wavelength of 734 nm in a UV spectrophotometer (Genesys 10S UV-Vis Spectrophotometer, Thermo Scientific, Waltham, MA, USA). As in the DPPH analysis, Trolox was used as a positive control, while deionized water was used as a blank control. The scavenging capacity (SC) was expressed as a percentage and was calculated according to Equation (3):

(3) $$\mathrm{SC}\left(\%\right)=\frac{\left(\mathrm{Ao}-\mathrm{Ai}\right)}{\mathrm{Ao}}\times 100$$

where Ao is the absorbance of ABTS^•+^ with solvent mixture, and Ai is the absorbance after reaction of ABTS^•+^ with the sample. SC(%) is the EO concentration that provided a 50% ABTS^•+^ scavenging effect.

### 4.10. Glucosidase Inhibition Activity

The inhibitory effect over the AG enzyme was determined according to the method report by Tao et al. [28] with some modifications detailed by Rengasamy et al. [29], as previously described by Morocho et al. [30]. The procedure was determined using pNPG as substrate. The essential oils were dissolved in DMSO at a final concentration of 10 mg/mL. Several dilutions in PBS were made to find the concentration that yields complete enzyme inhibition. In addition, 75 µL of PBS was mixed with 5 µL of the sample and 20 µL of the enzyme solution (0.15 U/mL in PBS pH 7.4); subsequently, the mixture was preincubated at 37 °C for 5 min prior to initiation of the reaction by adding the substrate. After preincubation, 20 µL of PNPG (5 mM in PBS, pH 7.4) were added and then incubated at 37 °C. The amount of p-nitrophenol released was measured in an EPOCH 2 (BIOTEK, Winooski, VT, USA) microplate reader every 5 min at 405 nm. Acarbose was put in place of the essential oils as a positive control. Inhibition concentration (IC) was expressed as a percentage and was calculated according to Equation (4):

(4) $$\mathrm{IC}\left(\%\right)=\frac{\left(\mathrm{Ao}\;\mathrm{As}\right)}{\mathrm{Ao}}\times 100$$

where Ao is the absorbance recorded for enzyme activity without inhibitor (control), and As is the absorbance recorded for enzyme activity in the presence of the inhibitor (sample). IC(%) is the EO concentration that provided 50% inhibition.

### 4.11. Statistical Analysis

The procedures of essential oil extraction, determination of physical properties, identification of chemical constituents, determination of antioxidant capacity, and α-glucosidase inhibition activity were repeated three times. The evaluation of antimicrobial activity was repeated nine times. The data were collected in Microsoft Excel, and the measures of central tendency were calculated using Minitab 17 (Version 17.1.0, Minitab LLC., State College, PA, USA). All results are expressed as mean values.

## 5. Conclusions

The chemical composition, physical properties, and antibacterial, antifungal, antioxidant, and antiglucosidase activity of the essential oil from *C. ferrugineus* leaves were determined. This study of the chemical composition and bioactivity of the essential oil from *C. ferrugineus* contributes to our knowledge concerning endemic species of Ecuador. Furthermore, with this study, it is intended to motivate detailed research studies of the essential oils from other endemic plant species. Motivated by the very strong antiglucosidase activity shown by the essential oil, other antienzymatic tests should be carried out on this oil, and the anticholinesterase activity can be proposed for further studies.

## Figures and Tables

**Table 1:** Chemical compounds present in the essential oil from *Croton ferrugineus* leaves.

No.	RT	Compound	RI	RI^ref^	*C. ferrugineus* EO		Type	CF	MM (Da)
					%	SD			
1	9.92	α-Thujene	925	924	0.35	0.14	ALM	C_10_H_16_	136.13
2	10.27	α-Pinene	933	932	2.31	0.24	ALM	C_10_H_16_	136.13
3	11.82	Sabinene	968	969	0.39	0.10	ALM	C_10_H_16_	136.13
4	12.04	β-Pinene	973	974	0.40	0.21	ALM	C_10_H_16_	136.13
5	12.74	Myrcene	989	988	11.47	1.56	ALM	C_10_H_16_	136.13
6	13.35	α-Phellandrene	1003	1002	0.86	0.48	ALM	C_10_H_16_	136.13
7	13.71	1,4-Cineole	1011	1012	0.35	0.01	OXM	C_10_H_18_O	154.14
8	14.06	ρ-Cymene	1019	1020	2.22	0.56	ARM	C_10_H_14_	134.10
9,10	14.29	Limonene + β-Phellandrene ^1^	1024	1024	14.88	1.27	ALM	C_10_H_16_	136.13
11	15.12	(E)-β-Ocimene	1043	1044	2.73	0.14	ALM	C_10_H_16_	136.13
12	15.61	γ-Terpinene	1054	1054	1.59	0.37	ALM	C_10_H_16_	136.13
13	17.33	Linalool	1093	1095	7.34	0.72	OXM	C_10_H_18_O	154.14
14	19.47	Camphor	1141	1141	0.58	0.06	OXM	C_10_H_16_O	152.12
15	21.61	Isoamyl tiglate	1190	1191	1.56	0.63	OXM	C_10_H_18_O_2_	170.13
16	26.02	2-Undecanone	1290	1293	3.52	0.49	OTC	C_11_H_22_O	170.17
17	28.37	α-Terpinyl acetate	1343	1346	0.41	0.11	OTC	C_12_H_20_O_2_	196.14
18	29.63	α-Ylangene	1372	1373	0.42	0.08	ALS	C_15_H_24_	204.19
19	30.12	trans-Myrtanol acetate	1383	1385	1.37	0.22	OTC	C_12_H_20_O_2_	196.14
20	31.53	trans-Caryophyllene	1415	1417	20.47	1.25	ALS	C_15_H_24_	204.19
21	32.33	γ-Elemene	1433	1434	0.62	0.28	ALS	C_15_H_24_	204.19
22	32.54	Aromadendrene	1438	1439	1.28	0.89	ALS	C_15_H_24_	204.19
23	33.08	α-Humulene	1450	1452	5.49	0.38	ALS	C_15_H_24_	204.19
24	33.56	9-epi-(E)-Caryophyllene	1461	1464	0.54	0.07	ALS	C_15_H_24_	204.19
25	34.31	Germacrene D	1478	1480	7.60	0.60	ALS	C_15_H_24_	204.19
26	35.10	Bicyclogermacrene	1496	1500	3.28	0.22	ALS	C_15_H_24_	204.19
27	35.83	γ-Cadinene	1513	1513	0.47	0.07	ALS	C_15_H_24_	204.19
28	36.11	β-Sesquiphellandrene	1519	1521	1.32	0.17	ALS	C_15_H_24_	204.19
29	36.20	δ-Cadinene	1521	1522	1.19	0.11	ALS	C_15_H_24_	204.19
30	37.79	Germacrene B	1557	1559	0.83	0.12	ALS	C_15_H_24_	204.19
31	38.58	Spathulenol	1575	1577	1.07	0.13	OXS	C_15_H_24_O	220.18
32	38.81	Caryophyllene oxide	1580	1582	0.79	0.19	OXS	C_15_H_24_O	220.18
33	41.45	epi-α-Cadinol	1640	1638	0.77	0.13	OXS	C_15_H_26_O	222.20
34	41.89	4-α-hydroxy-Dihydro agarofuran	1650	1651	0.72	0.19	OXS	C_15_H_26_O_2_	238.19
35	43.29	α-Bisabolol	1682	1685	0.77	0.20	OXS	C_15_H_26_O	222.20
Aliphatic monoterpene hydrocarbons (ALM)					34.98				
Aromatic monoterpene hydrocarbons (ARM)					2.22				
Oxygenated monoterpenes (OXM)					9.83				
Aliphatic sesquiterpene hydrocarbons (ALS)					43.50				
Oxygenated sesquiterpene (OXS)					4.13				
Other compounds (OTC)					5.30				
Total identified					99.95				

RT: Retention Time; RI: Calculated Retention Indices; RI^ref^: References Retention Indices; SD: Standard Deviation; CF: Chemical Formula; MM: Monoisotopic Mass. ^1^ Co-eluted compounds.

**Table 2:** Antimicrobial activity of the essential oil from *Croton ferrugineus*.

Microorganism	Essential Oil (µg/mL)	Positive Control ^a^ (µg/mL)
Gram-negative bacteria		
*Escherichia coli* (ATCC 43888)	>2500	1.56
Gram-positive bacteria		
*Enterococcus faecalis* (ATCC 19433)	>2500	1.95
*Micrococcus luteus* (10240)	2000	0.39
*Staphylococcus aureus* (ATCC 25923)	>2500	0.39
Dermatophyte fungi		
*Cándida albicans* (ATCC 10231)	1000	0.13

^a^ Tetracycline for *Enterococcus faecalis*, gentamicin for *Escherichia coli*, *Micrococcus luteus* and *Staphylococcus aureus*, and ketoconazole for fungus.

**Table 3:** Antioxidant activity and α-glucosidase inhibition properties of essential oils from *H. racemosum* leaves.

Sample	ABTS	DPPH	α-Glucosidase
	SC_50_ * (μg/mL)		IC_50_ * (μg/mL)
Essential oil	901 ± 20	>1000	146 ± 20
Trolox	420 ± 20	840 ± 30	–
Acarbose	–	–	70 ± 10

* SC_50_ = Scavenging capacity of 50%; IC_50_ = concentration required for 50% inhibition.

## Data Availability

Data are available from the authors upon reasonable request.

## Sample Availability

Samples of the *Croton ferrugineus* essential oil are available from the authors.
